# Numerical Simulation of Steel-Reinforced Reactive Powder Concrete Beam Based on Bond-Slip

**DOI:** 10.3390/ma14154176

**Published:** 2021-07-27

**Authors:** Haoxu Li, Xiao Guo, Jiqiang Duan

**Affiliations:** 1 College of Civil Engineering and Architecture, Guangxi University, Nanning 530004, China; haoxuli2020@163.com (H.L.); jiqiangduan@gxu.edu.cn (J.D.); 2 Key Laboratory of Disaster Prevention and Structural Safety of Chinese Ministry of Education, Guangxi University, Nanning 530004, China

**Keywords:** concrete damaged plasticity model, constitutive relationship, bond-slip, steel-reinforced reactive powder concrete beam, numerical simulation

## Abstract

In this study, based on the concrete damaged plasticity (CDP) model in the ABAQUS software, various plastic damage factor calculation methods were introduced to obtain CDP parameters suitable for reactive powder concrete (RPC) materials. Combined with the existing tests for the bending performance of steel-reinforced RPC beams, the CDP parameters of the RPC material were input into ABAQUS to establish a finite element model considering the bond and slip between the steel and RPC for numerical simulation. The load-deflection curve obtained by the simulation was compared with the measured curve in the experiment. The results indicated that on the basis of the experimentally measured RPC material eigenvalue parameters, combined with the appropriate RPC constitutive relationship and the calculation method of the plastic damage factor, the numerical simulation results considering the bond-slip were in good agreement with the experimental results with a deviation of less than 10%. Thus, it is recommended to select a gentle compressive stress-strain curve in the descending section, an approximate strengthening model of the tensile stress-strain curve, and to use the energy loss method and Sidoroff’s energy equivalence principle to calculate the RPC plastic damage parameters.

## 1. Introduction

The concrete damaged plasticity (CDP) model in the ABAQUS finite element software is widely used for the simulation analysis of the mechanical properties of ordinary concrete structural members. At present, several calculation methods for CDP model parameters of concrete have been proposed [1,2,3,4,5], including the calculation of concrete damage based on energy loss, specific strain method or graphic method, and a combination of elastic modulus damage and energy equivalence principle of Sidoroff. However, these methods utilize the CDP model parameters of normal strength concrete in ABAQUS. Although the method to calculate the CDP model parameters for ordinary concrete has been established, its applicability for reactive powder concrete (RPC) has not been studied. Thus, the calculation of the CDP model parameters for RPC is of utmost importance for the numerical simulation of RPC structures using ABAQUS software. However, only a few studies have been conducted on CDP model parameters for RPC [6,7,8]. In addition, a national standard for the RPC tension compression constitutive equation in China is lacking, which is an important factor for the calculation of CDP model parameters for RPC.

In this paper, we introduce several methods for the calculation of CDP model parameters in ABAQUS software. These methods were applied to calculate the CDP model parameters of RPC. The calculated parameters were compared and analyzed to determine their applicability. This paper reviews the constitutive relationship of RPC. Furthermore, the relationships were compared and classified to determine the appropriate constitutive relationship according to the strength characteristic value of RPC measured using existing tests. The calculated CDP model parameters were applied to the ABAQUS finite element software to simulate the bending resistance of steel-reinforced RPC beams. Comparing the results with the measured load-deflection curve, the rationality of the selected calculation method and RPC constitutive relationship was verified, thereby providing a reference for numerical analysis.

## 2. Parameter Calculation Method of Plastic Damage Model

### 2.1. Specific Strain Method or Graphic Method

**Figure 1:**
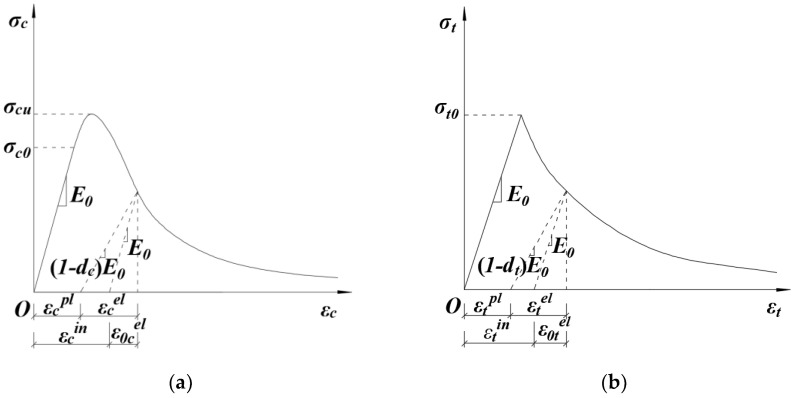
Uniaxial compression and tensile behaviour of concrete. (**a**) Stress-strain relationship of plastic damage under compression; (**b**) Stress-strain relationship of plastic damage under tension.

The definition of uniaxial compression and tension behavior of concrete in ABAQUS is depicted in Figure 1.

As shown in Figure 1, the expression of the stress-strain relationship of concrete under uniaxial compression and tension can be defined as follows:

(1) $$\sigma_{k}=(1-d_{k})E_{0}(\varepsilon_{k}-\varepsilon_{k}{}^{pl}),(k=t,c)$$

The damage factor in the above formula is calculated as follows:

(2) $$d_{k}=1-\frac{\sigma_{k}}{(\varepsilon_{k}-\varepsilon_{k}{}^{pl})E_{0}},(k=t,c)$$

where $\sigma_{k}$ represents tension stress, $\varepsilon_{k}{}^{}$ denotes tension strain, $\varepsilon_{k}{}^{pl}$ represents equivalent plastic strain, $\varepsilon_{k}{}^{in}$ represents inelastic strain, $E_{0}$ denotes the initial elastic modulus of the material.

Substituting $\varepsilon_{k}=\varepsilon_{k}{}^{in}+\varepsilon_{0k}{}^{el}$,$\varepsilon_{0k}{}^{el}=\sigma_{k}/E_{0}$ into (2), we obtain

(3) $$d_{k}=1-\frac{\sigma_{k}}{\left(\sigma_{k}+\varepsilon_{k}{}^{pl}(1/b_{k}-1)E_{0}\right)}$$

where k=t,c represents tension and compression, respectively; $b_{k}$ denotes the ratio of inelastic strain $\varepsilon_{k}{}^{in}$ to equivalent plastic strain $\varepsilon_{k}{}^{pl}$. According to the cyclic loading and unloading stress path of the concrete specimen, Birtel et al. [1] demarcated $b_{k}$ and suggested that $b_{c}=0.7$ and $b_{t}=0.7$. Zhang Jin et al. [2] suggested that the value of $b_{c}$ was within the range of 0.35–0.7 under compression and the value of $b_{t}$ was within the range of 0.5–0.95 under tension. The expression of equivalent plastic strain can also be obtained from Figure 1

(4) $$\varepsilon_{k}{}^{pl}=\varepsilon_{k}{}^{in}-\frac{d_{k}\sigma_{k}}{(1-d_{k})E_{0}}$$

The value of the equivalent plastic strain calculated by Equation (4) is expected to increase with an increase in the inelastic strain. Additionally, a negative value should not be obtained as the ABAQUS software will consider it as an error and terminate the calculation.

### 2.2. Energy Loss Method

According to the Najar damage theory [3], the uniaxial damage of brittle solid materials such as concrete can be defined as

(5) $$d=\frac{W_{0}-W_{\varepsilon }}{W_{0}}$$

where $W_{0}=\frac{E_{0}\varepsilon^{2}}{2}$ denotes the strain energy density function of a non-destructive material and $W_{\varepsilon }=\int \sigma d\varepsilon =\int f(\varepsilon )d\varepsilon$ denotes the strain energy density function of the material in a damaged state. $E_{0}$ denotes the initial elastic modulus of the material. In the Najar damage theory, the strain energy of the damaged state is obtained by linear simplification, $W_{\varepsilon }=\frac{\sigma \varepsilon }{2}$. However, the damage value calculated using this equation is larger than that of the actual value. When the stress-strain curve equation f(ε) of concrete is known, the damage factor of concrete under uniaxial loading is calculated accurately using a numerical integration method.

### 2.3. Combination of Elastic Modulus Damage and Energy Loss

Ding et al. [4] assumed that the complete curve of concrete under monotonic loading under uniaxial stress was consistent with the skeleton curve under cyclic loading and unloading. Furthermore, deformation of concrete occurs under uniaxial stress. In this case, the strain energy of concrete is denoted as W, and the irreversible deformation after unloading is caused by energy dissipation. The energy consumption is considered as $W_{I}$. The damage of concrete results in energy dissipation and stiffness reduction. The unloading stiffness of concrete can be defined as $(1-d_{1})E_{0}$ and $d_{1}$, which represent the damage variables of the elastic modulus. The energy loss variable $d_{2}$ can be defined as

(6) $$d_{2}=\frac{W_{I}}{W}=\frac{1-W_{\varepsilon }}{W}$$

where W=∫σdε=∫f(ε)dε; $W_{e}$ denotes the recoverable elastic strain energy, and $W_{e}=\frac{\sigma^{2}}{2(1-d_{1})E_{0}}$. To simplify the calculation, it is considered that the damage variables defined are equal in value. Hence, the damage variable can be defined as follows:

(7) $$d=d_{1}=d_{2}=1-\frac{\sigma }{\sqrt{2WE_{0}}}$$

### 2.4. Energy Equivalence Principle of Sidoroff

According to Sidoroff’s energy equivalence principle, the elastic residual energy generated by the stress acting on the damaged material is similar to that of the elastic residual energy produced by the stress acting on the non-destructive material. Therefore, the equivalent stress or elastic modulus is replaced in the equation with the elastic modulus at the time of damage [5].

The elastic complementary energy of an undamaged material is as follows:

(8) $$W_{0}{}^{e}=\frac{\sigma^{2}}{2E_{0}}$$

The elastic residual energy of the damaged material is as follows:

(9) $$W_{d}{}^{e}=\frac{\tilde{\sigma }}{2E_{0}}=\frac{\sigma^{2}}{2E_{d}}$$

Effective stress:

(10) $$\tilde{\sigma }=\frac{\sigma }{1-d}$$

Combining the abovementioned equations, we obtain $E_{d}={\left(1-d\right)}^{2}E_{0}$. Hence, the stress-strain relationship of the damaged material can be obtained by

(11) $$\sigma ={\left(1-d\right)}^{2}E_{0}\varepsilon$$

When the stress-strain relationship of concrete under uniaxial loading is known, the expression of the damage factor can be deduced by combining Equation (11).

### 2.5. Weibull Distribution Considering Damage Threshold

In [9], based on the continuous damage mechanics theory and statistical strength theory, the damage threshold parameter was introduced into the traditional two-parameter Weibull distribution function, and the damage evolution equation was derived as

(12) $$D=\left\{\begin{array}{l} 0,(\varepsilon <\gamma )\\ 1-\exp\left[-{\left(\frac{\varepsilon -\gamma }{\alpha }\right)}^{\beta }\right],(\varepsilon \geq \gamma ) \end{array}\right.$$

The damage constitutive relationship of concrete can be obtained by considering the effective stress after damage instead of the nominal stress:

(13) $$\sigma =(1-D)E_{0}\varepsilon$$

By substituting Equation (13) into Equation (12), the expression of the damage constitutive relationship of concrete can be obtained:

(14) $$\sigma =\left\{\begin{array}{l} E_{0}\varepsilon ,(\varepsilon <\gamma )\\ E_{0}\varepsilon \exp\left[-{\left(\frac{\varepsilon -\gamma }{\alpha }\right)}^{\beta }\right],(\varepsilon \geq \gamma ) \end{array}\right.$$

According to the boundary conditions of the concrete stress-strain curve, the stress-strain value at the beginning of the curve is zero, and the slope at the top of the curve is zero:

(15) $$\beta =\frac{\varepsilon_{pk}-\gamma }{\varepsilon_{pk}\ln\frac{E_{0}\varepsilon_{pk}}{\sigma_{pk}}},\alpha =\frac{\varepsilon_{pk}-\gamma }{{\left(\frac{\varepsilon_{pk}-\gamma }{\beta \varepsilon_{pk}}\right)}^{\frac{1}{\beta }}}$$

In Equation (15), $\varepsilon_{pk}$ and $\sigma_{pk}$ denote the peak strain and stress on the stress-strain curve of concrete, respectively. According to the mechanical tests conducted on ordinary concrete, fiber-reinforced concrete, and ultra-high performance concrete, the elastic limit strain is $\gamma \approx (0.7∼0.9)\varepsilon_{pk}$. According to [8], the damage variable D in Equation (12) is different from that of the definition of the damage factor of the CDP model parameters in ABAQUS. Therefore, it cannot be directly input into the software. However, the value of D was directly input into the software in [6].

### 2.6. Parameter Identification Method Based on Test Data

In [7], according to the damage test, the damage factor of ordinary concrete and the normalised inelastic strain follow the first-order exponential decay function:

(16) $$d_{k}=A_{0}e^{\left(-\frac{\varepsilon_{norm}{}^{in}}{t_{0}}\right)}+B_{0}$$

where t,c represent tension and compression respectively, and $\varepsilon_{norm}{}^{in}$ represents the normalised inelastic strain. Since Equation (16) passes through two points (0,0) and (1,1), $A_{0}$, $B_{0}$, and $t_{0}$ can be represented by an unknown quantity. If $t_{0}$ is used to represent $A_{0}$ and $B_{0}$, then $A_{0}=\frac{1}{e^{-\frac{1}{t_{0}}}-1},B_{0}=-\frac{1}{e^{-\frac{1}{t_{0}}}-1}$. The physical meaning of $t_{0}$ implies the evolution rate of the damage factor with inelastic strain. According to the test data in [7], $t_{0}=0.65$ is considered appropriate. To establish a general law for damage factor evolution, the relationship table of damage factor, strain ratio, and stress ratio was arranged by assuming that there exists a one-to-one correspondence between the damage factor and stress ratio or strain ratio.

## 3. Constitutive Relationship of RPC under Uniaxial Tension and Compression

### 3.1. Stress-Strain Relationship under Uniaxial Compression

Various constitutive equations have been established based on the uniaxial compression tests of ultra-high performance concrete. The ascending part of the stress-strain curve under uniaxial compression can be classified into a straight line [10,11,12], a polynomial curve, a rational fraction curve, and an exponential curve. It is an empirical approach, based on a good description of experimental data. But excepting the first part, without a certain model to sustain them, only the Ax term obeys to HOOK’s law.

#### 3.1.1. Ascending Part of the Curve as a Polynomial

In [13], based on the clear physical quantity of the elastic modulus, the ascending part of the stress-strain curve of RPC under uniaxial compression was defined as a polynomial consisting of only one parameter, while the descending part of the curve was fitted with a rational fraction according to the test data:

(17) $$y=\left\{\begin{array}{l} Ax+Bx^{a}+Cx^{b},(0\leq x\leq 1)\\ \frac{x}{\alpha {\left(x-1\right)}^{2}+x},(x\geq 1) \end{array}\right.$$

where $x=\varepsilon_{c}/\varepsilon_{c0}$,$\varepsilon_{c}$ denotes the strain of concrete under compression, and $\varepsilon_{c0}$ denotes the peak strain; $y=\sigma_{c}/f_{c}$,$\sigma_{c}$ denotes the compressive stress of concrete and $f_{c}$ denotes the axial compressive strength of the prism. The parameter $A=E_{0}\varepsilon_{c0}/f_{c}$ denotes the ratio of the initial elastic modulus $E_{0}$ to the secant modulus $E_{p}$ at the peak point. In the following, x,y, and A have the same meaning, and α is a parameter in the descending section. B=5−4A,a=4,C=3A−4,b=5 in [13]; B=6−5A,a=5,C=4A−5,b=6 in [14].

#### 3.1.2. Ascending Part of the Curve as a Rational Fraction

Based on the expression of the stress-strain curve of the steel-fiber-reinforced high-strength concrete, [15] simplified the stress-strain curve according to the characteristics of the entire stress-strain curve. After simplification, the ascending part of the stress-strain curve applicable to RPC is given by Equation (18), and the descending part is the same as that in [13].

(18) $$y=\frac{Ax-x^{2}}{1+(A-2)x},(0\leq x\leq 1)$$

The constitutive equation of ultra-high performance concrete under uniaxial compression proposed in [16] is the same as that in [15], and the descending part is shown in Formula (19), where $B=0.25{\left(0.125A\sqrt{{\left(0.125A+0.8\right)}^{2}-0.8}-0.2\right)}^{1.5}$.

(19) $$y=\frac{1}{1+B{\left(x-1\right)}^{1.5}},(x\geq 1)$$

#### 3.1.3. Ascending Part of the Curve as an Exponential

Considering the experiment conducted in [17], only the ascending part of the curve was measured, and different functions were used for fitting based on the least squares principle. The fitting effect of the exponential function was found to be the best. Therefore, only the fitting function expression of the ascending part of the curve is given:

(20) $$y=Ax(1.000176-0.000176e^{\frac{x}{0.174}})$$

**Figure 2:**
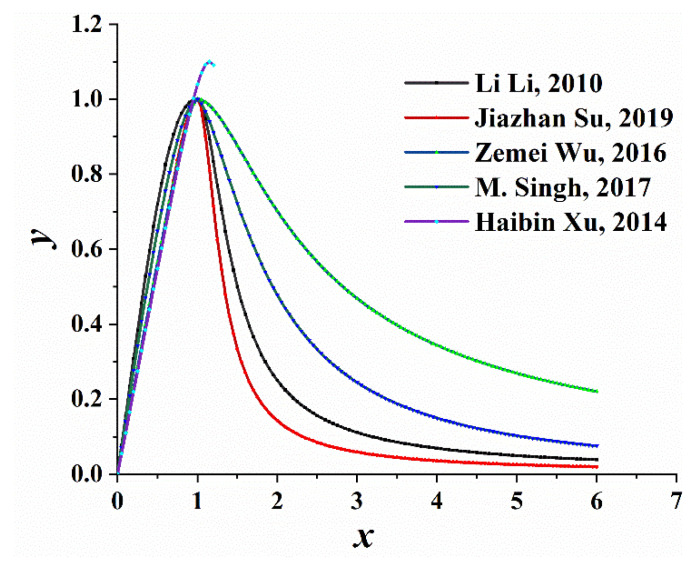
Uniaxial compressive stress-strain curve.

The above compression constitutive curve was drawn on a graph, as illustrated in Figure 2. In the ascending part of the curve, the larger the parameter value, the steeper the stress-strain curve and the shorter the elastic section; During parameter selection, the proportion, maintenance conditions, and physical characteristic values of the test block, such as peak value, limit value, and elastic modulus should be considered. The descending part of the curve fluctuates significantly because the data are complex to measure during the test and are considerably affected by the test conditions. The actual data of the test should be fitted while selecting the parameters.

### 3.2. Stress-Strain Relationship under Uniaxial Tension

Based on the uniaxial tensile test data of the RPC test block, in combination with the theoretical analysis, Li et al. [13] uses two equations, which are continuous at the peak point, to describe the two equations respectively:

(21) $$y=\left\{\begin{array}{l} Ax+(3-2A)x^{2}+(A-2)x^{3},(0\leq x\leq 1)\\ \frac{x}{\alpha {\left(x-1\right)}^{\beta }+x},(x\geq 1) \end{array}\right.$$

where $x=\varepsilon_{t}/\varepsilon_{t0}$,$\varepsilon_{t}$ denotes the tensile strain of concrete, and $\varepsilon_{t0}$ denotes the peak strain; $y=\sigma_{t}/\sigma_{t0}$,$\sigma_{t}$ denotes the tensile stress of concrete and $\sigma_{t0}$ denotes the axial tensile strength of the prism. Parameter $A=E_{0}\varepsilon_{t0}/f_{t}$ denotes the ratio of the initial modulus of elasticity $E_{0}$ to the secant modulus $E_{p}$ at the peak point, and α is a parameter in the descending part of the curve, among which α=5.5,β=2.2.

Based on the experimental results, the stress-strain relationship proposed in [18] is as follows:

(22) $$\sigma (\varepsilon )=\left\{\begin{array}{l} \frac{f_{ct}}{\varepsilon_{ca}}\varepsilon ,(0\leq \varepsilon \leq \varepsilon_{ca})\\ f_{ct},(\varepsilon_{ca}\leq \varepsilon \leq \varepsilon_{cp}) \end{array}\right.$$

where $\varepsilon_{ca}$ denotes the linear deviation from the initial crack strain, $\varepsilon_{cp}$ denotes the ultimate strain, and $f_{ct}$ denotes the average stress in the strain hardening stage.

The authors of [19] provide a micro-scale calculation method for residual tensile strength of ultra-high performance concrete, which can consider fiber distribution, buried depth, and orientation. The tensile stress-strain relationship is as follows:

(23) $$\sigma_{t}=\left\{\begin{array}{l} E_{0}\varepsilon ,(\varepsilon \leq \varepsilon_{p})\\ 0.304\sqrt{f_{cu}}\frac{\rho_{f}l_{f}}{d_{f}},(\varepsilon_{p}<\varepsilon <\varepsilon_{t\lim}) \end{array}\right.$$

where $\varepsilon_{p}=f_{t}/E_{0}$ denotes the ultimate elastic strain and $\varepsilon_{t\lim}$ denotes the ultimate tensile strain, considering 0.01; $d_{f}$ denotes the fiber diameter, $\rho_{f}$ denotes the fiber volume content, and $l_{f}$ denotes the fiber length.

In [20], the expression of the uniaxial tensile stress-strain relationship of ultra-high performance concrete is as follows:

(24) $$y=\left\{\begin{array}{l} \frac{Ax}{Bx^{C}+1},(0\leq x\leq 1)\\ \frac{x}{\alpha {\left(x-1\right)}^{\beta }+x},(x\geq 1) \end{array}\right.$$

where B=A−1,$C=\frac{A}{A-1}$, parameters of the descending part α=0.025,β=1.7.

The expression of uniaxial tension relation mentioned in [14] is as follows:

(25) $$y=\left\{\begin{array}{l} \frac{x}{0.92x^{1.09}+1},(0\leq x\leq 1)\\ \frac{x}{0.1{\left(x-1\right)}^{2.4}+x},(x\geq 1) \end{array}\right.$$

**Figure 3:**
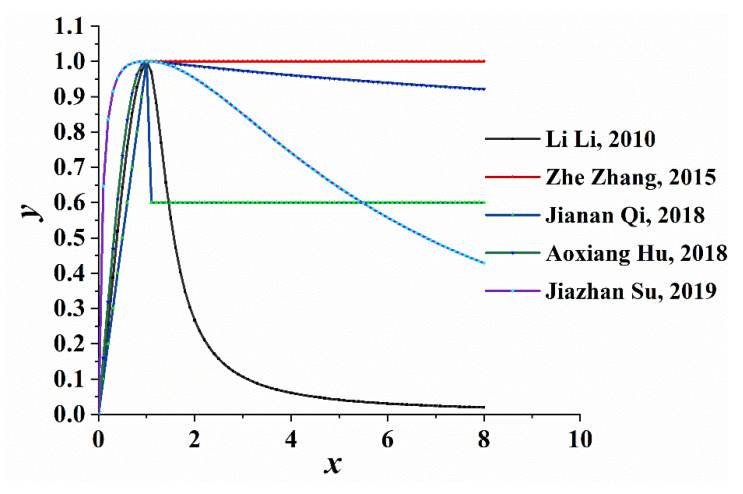
Uniaxial tensile stress-strain curve.

Based on the tensile constitutive relationship curve depicted in Figure 3, it can be observed that the ascending part of the curve resembles a straight line, which is in good agreement with the measured data in the literature. Similar to the uniaxial tensile stress-strain constitutive relationship, the larger the value of A, the steeper the ascending part of the curve and the shorter the linear elastic section. The descending part of the curve varies significantly in different literature, reflecting the strain strengthening and softening. The descending part of the curve can be smoothed by adjusting the descent parameters.

## 4. Experimental Simulation of Steel-Reinforced Reactive Powder Concrete Beams under Bending

### 4.1. Test Data Reference

**Figure 4:**
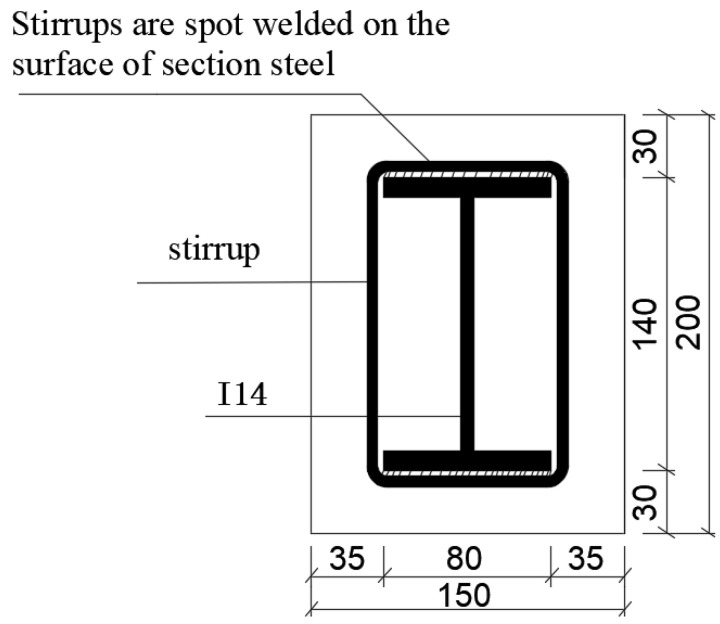
Size of beam cross-section (unit: mm).

The three-point bending test of the SRC-1 steel-reinforced RPC beam in [21] was selected for the numerical simulation. The length of the test beam was 2000 mm, and the section size was B × h = 150 × 200 mm. The effective span was 1800 mm. A Q235-B section steel was used for the built-in I-beam, and the I-beam specification was I14. The section steel length was 1950 mm, which was placed in the middle of the beam, and the thickness of the protective layer was 30 mm. HPB300 grade reinforcement was used for the stirrups, which were spot-welded on the surface of the section steel. The spacing of the stirrups in the pure bending section was 200 mm, and the spacing of the other parts was 100 mm. The cross-section of the test beam is illustrated in Figure 4, and the material performance characteristic values are presented in Table 1.

### 4.2. Constitutive Relationship of Materials

In general, the brittleness of concrete increases with its strength. However, the addition of fiber into RPC leads to ultra-high strength and high toughness. Therefore, based on the abovementioned uniaxial tension compression constitutive relationship curve of RPC, the gentle descending section of the constitutive relationship curve was selected. In particular, the descending part of the uniaxial tension constitutive relationship curve was selected. If the tensile deformation capacity is poor, it does not match with the ultra-high compressive strength. Therefore, the RPC in the tension zone cracks, and the deformation is significantly large. However, if the RPC in the compression zone does not reach the peak stress, the section steel buckles locally, making the calculation divergent. In this study, the constitutive relationship of uniaxial compression in [13] was selected. The uniaxial tension relationship curve represents an approximate strengthening model, and the constitutive relationship curve mentioned in [14] or [20] can be used. Therefore, the convergence of the calculation is good. In this study, the relationship curve in [14] was selected. According to the measured material parameters in Table 1 and the conversion formula of the RPC basic mechanical property index mentioned in [22], the corresponding peak strain and ultimate strain can be obtained. The tensile constitutive equation is given by Equation (25), and the concrete expression of the compressive constitutive equation is shown in Equation (26):

(26) $$y=\left\{\begin{array}{l} 1.28x-0.12x^{4}-0.16x^{5},(0\leq x\leq 1)\\ \frac{x}{8{\left(x-1\right)}^{2}+x},(x\geq 1) \end{array}\right.$$

**Figure 5:**
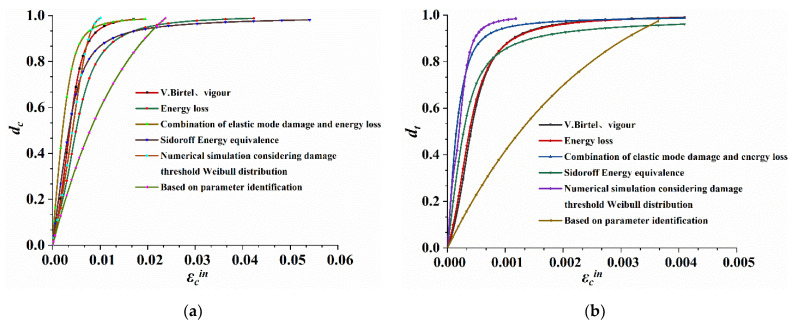
Damage factor and inelastic strain. (**a**) Compression damage factor inelastic strain. (**b**) Tension damage factor inelastic strain.

Comparing the six methods used to calculate the CDP model parameters in ABAQUS with the parameters calculated by Equations (25) and (26), as shown in Figure 5, this study uses the complex Simpson quadrature formula to obtain the damage factor.

**Figure 6:**
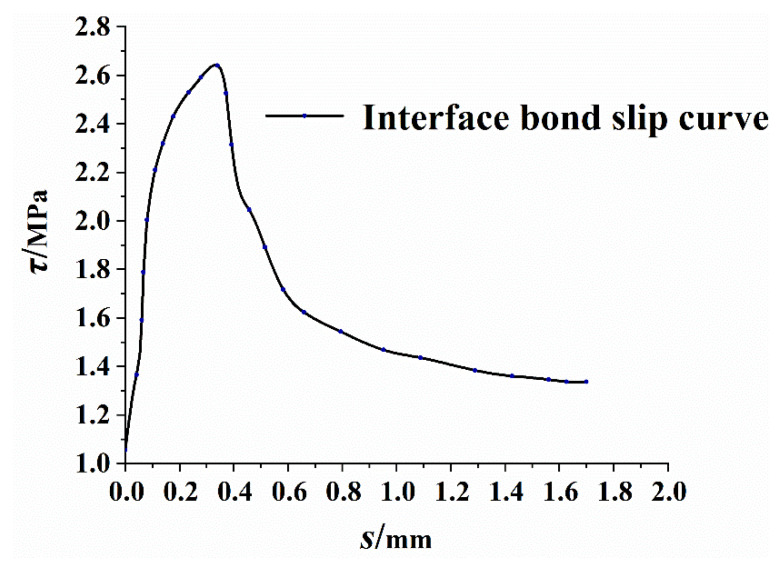
Interface bond strength-slip relationship (Bu Liang-tao, 2018).

The bilinear strengthening model was adopted for the section steel, and the elastic-plastic material properties input into the ABAQUS software are listed in Table 2. The bond-slip constitutive relationship between the section steel and RPC is based on the interface bond constitutive relationship adjusted in [23]; the discrete element method (DEM) is used, as is the bond-slip method in modeling with CDP in ABAQUS. According to the characteristic value of the interface bond strength, the relationship curve of the interface bond stress and slip was obtained, as depicted in Figure 6.

### 4.3. Establishment of Finite Element Model

**Figure 7:**
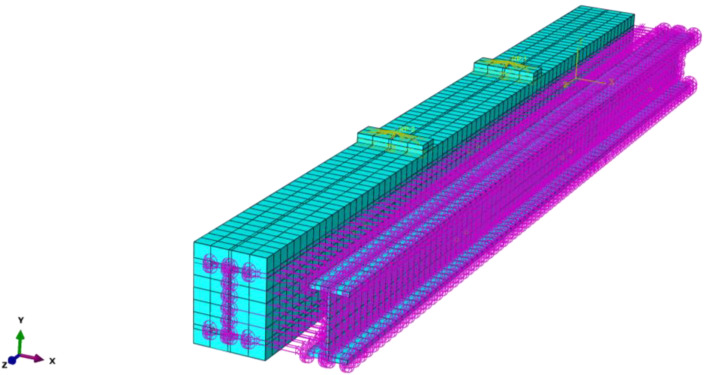
Finite element model of SRC beam.

The finite element model of the test beam is depicted in Figure 7. A rigid cushion block was set at the loading point to prevent the convergence due to stress concentration. The constraint between the stirrup and RPC was embedded, and the bond-slip was neglected. Spring 2 elements in three directions were used to simulate the interaction between the stirrup and RPC. The spring stiffness in the longitudinal and tangential direction were calculated according to the relationship curve shown in Figure 6, and the force displacement curve of the spring was obtained according to the surface area of each spring. The normal longitudinal direction considered that the two were completely bonded. The stiffness setting was larger, which was equal to the elastic modulus of the section steel or RPC, and the transverse tangential spring stiffness was set to half that of the longitudinal tangential spring stiffness. The elastic modulus of the rigid cushion block was set to 10 times that of the section steel. The rigid cushion block was bound with RPC, and two reference points were established above the cushion block to apply the displacement load. The C3D8R linear reduced integral element was used for the RPC, section steel, and rigid block, whereas the T3D2 element was used for the stirrup simulation.

**Figure 8:**
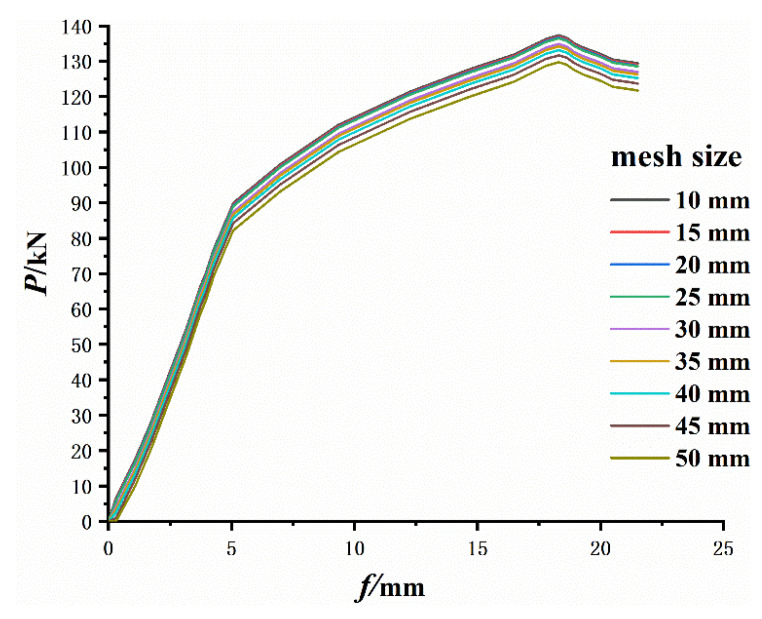
Load-deflection curve of different mesh size.

The mesh size in the cross-section is fixed, while the longitudinal mesh sizes were set to 10mm, 15 mm, 20 mm, 25 mm, 30 mm, 35 mm, 40 mm, 45 mm, 50 mm, respectively. The results calculated by the FE proposed are shown in Figure 8. It can be seen from Figure 8 that the influence of the mesh size on the results is very slight, and the peak load decreases slightly as the mesh size increases. When the mesh size increases from 10 mm to 15 mm, 20 mm, 25 mm, 30 mm, 35 mm, 40 mm, 45 mm and 50 mm, the maximum load decreases by 0.29%, 0.51%, 0.65%, 1.82%, 2.32%, 3.06%, 4.15% and 5.60%, respectively. It should be recognized that mesh sizes that are too small will result in high computational costs, while mesh sizes that are too large will result in lower sensitivity. The mesh size is related to many factors. Therefore, the mesh number and size should be determined according to the specific problem.

### 4.4. Comparative Analysis of Results

**Figure 9:**
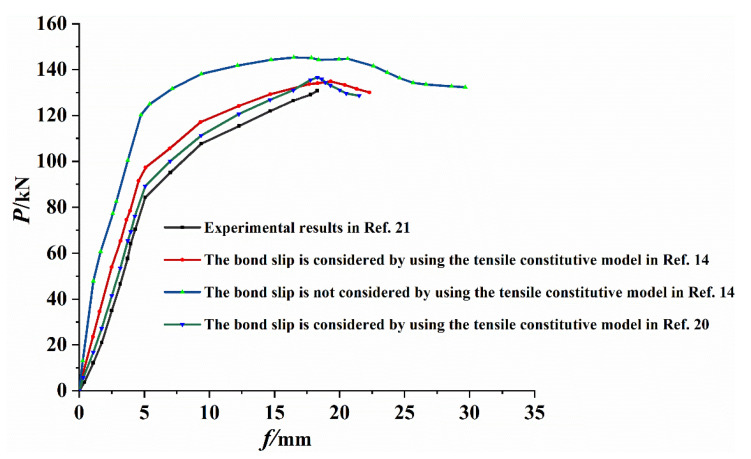
Load-deflection curve of numerical simulation and test results.

The load-deflection curve, which could appropriately represent the flexural performance of the test beam, was selected, and the finite element simulation results of the tensile constitutive model in [14] with and without bond-slip were obtained, and the simulation results of the tensile constitutive model in [20] considering bond-slip were compared with the test results, as shown in Figure 9 and Table 3. It can be observed from the figure that the results of the tensile constitutive model in [14,20] considering the bond-slip between the steel and RPC were in good agreement with the experimental results. The difference between the simulation results and test values was large, cracking load was large, error was 24.3%, ultimate bearing capacity error was 10.7%, stiffness of the entire simulation process was large, and ultimate displacement error was 10%. Moreover, the convergence of the model was good. However, there was an error between the results with or without the bond-slip and the experimental values due to the difference between the RPC constitutive relationship curve and the actual value. The dilatancy angle, eccentricity, the ratio of initial equibiaxial compressive yield stress to initial uniaxial compressive yield stress, the ratio of the second stress invariant on the tensile meridian, and viscosity coefficient were set as 30°, 0.1, 1.16, 0.6667 and 0.0005, respectively [24,25,26,27,28,29,30,31,32,33,34,35,36]. Thus, the bond-slip constitutive relationship between the section steel and RPC was not accurate.

To verify the consistency between the ultimate load of test and finite element simulation, the ultimate load ratio was calculated, as depicted in Table 3. It could be revealed that the average value was 0.94, the standard deviation was 0.031, and the coefficient of variation was 0.033. The numerical simulation results were in good agreement with the experimental results.

**Figure 10:**
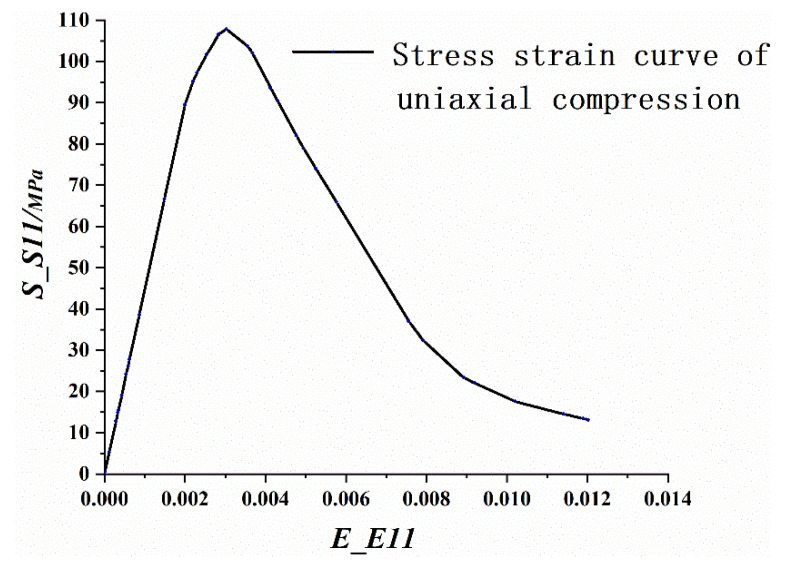
Longitudinal stress-strain at the edge of the midspan compression zone.

**Figure 11:**
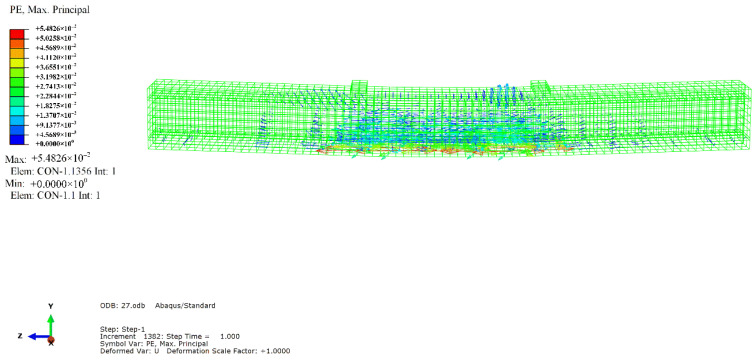
Maximum plastic strain cloud diagram and crack direction.

**Figure 12:**
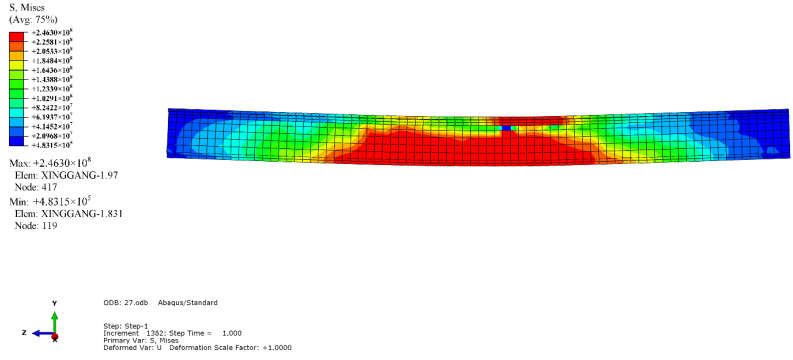
Von-Mises stress diagram of section steel.

**Figure 13:**
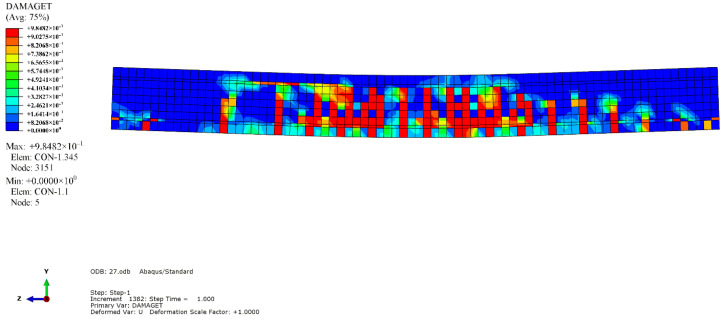
RPC tensile damage factor cloud diagram.

**Figure 14:**
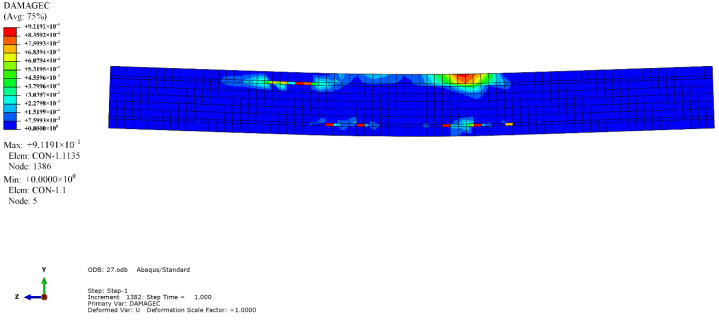
RPC compression damage factor cloud diagram.

The simulation results revealed that the mode of failure of the beam was bending failure. When the beam was damaged, the value of the compression strain reached 4500, which was on the edge of the compressive zone of RPC in the pure bending section. The stress-strain curve of the maximum longitudinal compressive strain point in the compressive zone of the midspan was extracted, as depicted in Figure 10. When the tensile equivalent plastic strain and maximum principal plastic strain of a point were positive, an initial crack occurred at that point, and the normal vector of the crack surface was parallel to the direction of the maximum plastic strain. As illustrated in Figure 11, the cracks were concentrated in the pure bending section in the middle of the span, forming three main cracks, with one inclined crack in each bending shear section on both sides. The tension flange and the webs of the section steel yielded under tension, and the compression flange yielded under local compression, as depicted in Figure 12. Figure 13 and Figure 14 reflect the tensile and compressive damage degrees, respectively, when the finite element simulation beam approaches the ultimate load.

## 5. Conclusions

(1) The energy loss method was suitable for all types of concrete materials and exhibited a high accuracy. Furthermore, the Sidoroff energy equivalence principle demonstrated good applicability. In this study, the energy loss method and Sidoroff energy equivalent principle were used to calculate the CDP model parameters of RPC materials in ABAQUS.
(2) The ascending part of the constitutive relationship curve of RPC and other ultra-high performance concrete under uniaxial compression adopted polynomial, rational fraction, and exponential forms. There is a minor difference among these forms, and the ascending part parameters have obvious physical meanings. However, the parameters of the descending part of the curve were significantly affected by the mix proportion, test equipment, and maintenance conditions. Therefore, there were obvious differences, which were obtained by fitting the test data. The uniaxial tension constitutive model could be classified into strain-softening, strain-hardening, and approximate strain-hardening types. Owing to the fiber crack resistance and fiber bearing tension, RPC exhibited good toughness and energy dissipation capacity. Thus, it is suggested that when the compressive constitutive model is selected, the value of parameters in the descending part of the curve should result in a gradual decline in the curve. Additionally, when the tensile constitutive model is selected, the approximate strain hardening model should be adopted.
(3) The numerical simulation results considering the bond-slip were in good agreement with the experimental results with a deviation of less than 10%, which verified the validity of the selected RPC constitutive relationship, and the CDP parameter calculation method was suitable for RPC materials. The numerical simulation performed in this study can provide a reference for the future use of ABAQUS software to simulate the mechanical properties of RPC and ultra-high performance concrete.

## Figures and Tables

**Table 1:** Material performance parameters (unit: MPa).

Section steel	*f_y_*	246
	*f_u_*	392
	*Es*	2.06 × 10^5^
RPC	*f_cu_*	127.3
	*f_c_*	113.3
	*f_ts_*	8.31
	*Ec*	4.7 × 10^4^

**Table 2:** Parameters of section steel.

Elastic Model/MPa	Poisson’s Ratio	Yield Stress/MPa	Inelastic Strain
206369.1	0.3	246.3	0
		399.5	0.0169

**Table 3:** Comparison of finite element simulation results and test results.

Model	Cracking Load/kN	Yield Load /kN	Ultimate Load/kN	Ultimate Deflection/mm	Ultimate Load Ratio
Test	62.2	108.0	131	18.13	/
Sliding is considered in reference [14]	65.3	117.1	134.8	19.3	0.97
Sliding is not considered in reference [14]	77.3	124.9	145.3	16.5	0.90
Sliding is considered in reference [20]	65.4	111.3	136.6	18.3	0.96

## Data Availability

The testing and analysis data used to support the findings of this study are included within the article.

## References

[1] Birtel V., Mark P. Parameterized finite element modelling of RC beam shear failure. Proceedings of the ABAQUS Users’ Conference.

[2] Zhang J., Wang Q., Hu S. (2008). Parameter verification of ABAQUS concrete damage plasticity model. Build. Struct..

[3] Wang Z., Yu Z. (2004). Concrete damage model based on energy loss. J. Build. Mater..

[4] Ding F., Yu Z., Ou J. (2008). Constitutive model of concrete uniaxial stress damage. J. Chang. Univ. (Nat. Sci. Ed.).

[5] Xiao Y., Chen Z., Zhou J., Leng Y., Xia R. (2017). Concrete plastic-damage factor for finite element analysis: Concept, simulation, and experiment. Adv. Mech. Eng..

[6] Zhu H. (2008). Plastic Damage Analysis of RPC Materials under Uniaxial and Biaxial Compressive Stress States.

[7] Li H. (2009). Parameter Identification and Finite Element Verification of Plastic Damage Constitutive Model of RPC Materials.

[8] Yang K., Sun L., Li W., Chen L., Xie Z. (2016). Sectional design and mechanical properties of RPC hollow compression members. Eng. Mech..

[9] Xue Y., Li S., Lin F., Xu H.B. (2009). Research on the damage constitutive model of steel fiber concrete considering the influence of damage threshold. Rock Soil Mech..

[10] Yoo D.-Y., Yoon Y.-S. (2015). Structural performance of ultra-high-performance concrete beams with different steel fibers. Eng. Struct..

[11] Chen S., Zhang R., Jia L.-J., Wang J.-Y. (2018). Flexural behaviour of rebar-reinforced ultra-high-performance concrete beams. Mag. Concr. Res..

[12] Yan J.-B., Hu H., Wang T. (2020). Flexural behaviours of steel-UHPC-steel sandwich beams with J-hook connectors. J. Constr. Steel Res..

[13] Li L. (2010). Research on the Mechanical Properties and Design Methods of Reactive Powder Concrete Beams.

[14] Su J.-Z., Ma X.-L., Chen B.-C., Sennah K. (2019). Full-scale bending test and parametric study on a 30-m span prestressed ultra-high performance concrete box girder. Adv. Struct. Eng..

[15] Wu Z., Shi C., He W., Wu L. (2016). Effects of steel fiber content and shape on mechanical properties of ultra high performance concrete. Constr. Build. Mater..

[16] Singh M., Sheikh A.H., Mohamed Ali M.S., Visintin P., Griffith M.C. (2017). Experimental and numerical study of the flexural behaviour of ultra-high performance fibre reinforced concrete beams. Constr. Build. Mater..

[17] Xu H., Deng Z. (2014). Experimental study on flexural performance of prestressed ultra-high performance steel fiber concrete beams. J. Build. Struct..

[18] Zhang Z., Shao X., Li W., Zhu P., Chen H. (2015). Axial tensile performance test of ultra-high performance concrete. China J. Highw. Transp..

[19] Qi J., Wang J., Ma Z.J. (2017). Flexural response of high-strength steel-ultra-high-performance fiber reinforced concrete beams based on a mesoscale constitutive model: Experiment and theory. Struct. Concr..

[20] Hu A., Liang X., Yu J., Shi Q., Li L. (2018). Experimental study on the mechanical properties of ultra-high performance concrete under axial tension. J. Hunan Univ. (Nat. Sci. Ed.).

[21] Liu D. (2017). Outsourcing Type Reactive Powder Concrete Beam Bending Performance Test Research.

[22] Lv X., Wang Y., Fu C., Zheng W. (2014). Reactive powder concrete basic mechanics performance index value. J. Harbin Inst. Technol..

[23] Bu L., Luo K. (2018). Steel outsourcing the stick performance of reactive powder concrete (RPC). Sci. Technol. Eng..

[24] Huang Z. (2018). Experimental Study and Optimal Design of UHPC Beam Model.

[25] Jian T. (2019). Research on Mechanical Behavior of UHPC Sandwich Floor.

[26] Liu B., Bai G.L., Xu Z.H., Ma J.F., Han Y.Y. (2019). Experimental study and finite element modelling of bond behavior between recycled aggregate concrete and the shaped steel. Eng. Struct..

[27] Bai L., Zhang M., Yang L., Hu S., Bao Z. (2021). Experimental study and finite element analysis on bond properties of high ductility steel reinforced cementitious materials. Eng. Mech..

[28] (2014). ABAQUS Theory Manual.

[29] Chou C.-C., Wu S.-C. (2019). Cyclic lateral load test and finite element analysis of high-strength concrete-filled steel box columns under high axial compression. Eng. Struct..

[30] Nasiri E., Liu Y. (2019). The out-of-plane behaviour of concrete masonry infills bounded by reinforced concrete frames. Eng. Struct..

[31] Farzad M., Shafieifar M., Azizinamini A. (2019). Experimental and numerical study on bond strength between conventional concrete and Ultra High-Performance Concrete (UHPC). Eng. Struct..

[32] Rabi M., Cashell K.A., Shamass R. (2019). Flexural analysis and design of stainless steel re-inforced concrete beams. Eng Struct.

[33] Bypour M., Gholhaki M., Kioumarsi M., Kioumarsi B. (2019). Nonlinear analysis to investigate effect of connection type on behavior of steel plate shear wall in RC frame. Eng. Struct..

[34] Xiao J. (2008). Recycled Aggregate Concrete.

[35] Liu H., Wang Y., He M., Shi Y., Waisman H. (2015). Strength and ductility performance of concrete-filled steel tubular columns after long-term service loading. Eng. Struct..

[36] Mirzazadeh M.M., Green M.F. (2017). Non-linear finite element analysis of reinforced concrete beams with temperature differentials. Eng. Struct..

